# Influence of substrate design for in vitro mechanical testing

**DOI:** 10.4317/jced.55353

**Published:** 2019-02-01

**Authors:** Amanda-Maria-de Oliveira Dal Piva, João-Paulo-Mendes Tribst, Alexandre-Luiz-Souto Borges, Renata-Marques de Melo, Marco-Antonio Bottino

**Affiliations:** 1 DDs, MSc, PhD Student, Department of Dental Materials and Proshodontics, São Paulo State University (Unesp), Institute of Science and Technology, São José dos Campos / SP, Brazil. Address: Av Engenheiro Francisco José Longo, 777, Jardim São Dimas, São José dos Campos, São Paulo, Brazil. CEP 12245-000. Department of Dental Materials Science, Academic Centre for Dentistry Amsterdam (ACTA), Universiteit van Amsterdam and Vrije Universiteit, Gustav Mahlerlaan #3004, 1081 LA Amsterdam, Noord-Holland, The Netherlands; 2 DDs, MSc, PhD, Adjunct Professor, Department of Dental Materials and Proshodontics, São Paulo State University (Unesp), Institute of Science and Technology, São José dos Campos / SP, Brazil. Address: Av Engenheiro Francisco José Longo, 777, Jardim São Dimas, São José dos Campos, São Paulo, Brazil. CEP 12245-000; 3 DDs, MSc, PhD, Researcher III, Department of Dental Materials and Proshodontics, São Paulo State University (Unesp), Institute of Science and Technology, São José dos Campos / SP, Brazil. Address: Av Engenheiro Francisco José Longo, 777, Jardim São Dimas, São José dos Campos, São Paulo, Brazil. CEP 12245-000; 4 DDs, MSc, PhD, Professor, Department of Dental Materials and Proshodontics, São Paulo State University (Unesp), Institute of Science and Technology, São José dos Campos / SP, Brazil. Address: Av Engenheiro Francisco José Longo, 777, Jardim São Dimas, São José dos Campos, São Paulo, Brazil. CEP 12245-000

## Abstract

**Background:**

The goal of this study was to evaluate the influence of dental substrate simulator material, and the presence of root and periodontal ligament on the stress distribution in an adhesively-cemented monolithic crown.

**Material and Methods:**

Five (5) 3D models according to the substrate simulator material and shape were modeled with CAD software for conducting non-linear finite element analysis (FEA): Tooth with and without periodontal ligament - subgroup “pl” (groups Tooth+pl and Tooth-pl), machined tooth in epoxy-resin with and without pulp chamber - subgroup “pc” (ER+pc and ER-pc) and simplified epoxy-resin substrate without pulp chamber and roots (SiER). Next, adhesively-cemented monolithic crowns in zirconia reinforced lithium silicate were modeled over each substrate. The solids were then imported in STEP format to the analysis software and the contact between teeth and cylinder was considered perfectly bonded; whereas, the contacts involving the resin cement were considered as non-separated. The materials were considered isotropic, linearly elastic, and homogeneous. An axial load (600 N) was applied to the occlusal surface and results of maximum principal stress (MPa) on the restoration were required.

**Results:**

FEA revealed that all evaluated subtracts showed the crown intaglio surface as the most stressed region. The average stress and stress peaks were similar for restorations cemented onto Tooth+pl, Tooth-pl and ER+pc substrates, but, 13% higher in comparison to ER-pc and SiER substrates.

**Conclusions:**

Simplified substrates can be used to evaluate posterior full crown behavior without periodontal ligaments and roots, since the rigidity of the specimen is taken into account.

** Key words:**Finite element analysis, axial loading, computed assisted numerical analisys, monolithic crowns,methodological study.

## Introduction

The field of materials science advances in developing new materials that often require new research to predict their behavior in use. Among dental materials, ceramics are widely used due to high aesthetics and mechanical properties (1). Thus, in respecting their limitations and following correct clinical application, ceramic restorations present promising results with high survival rates (2,3).

It is quite common for dental materials to be released on the market prior to clinical trials. Therefore, in searching for relevant information and short-term results, companies and researchers use laboratory studies which are most often fatigue tests (4-11). But when an experimental study is performed, several factors must be taken into account such as sample design, testing methods, data analysis and possible outcomes (4). Thus, several guides have been launched in order to standardize and guide researchers in developing sound studies that can be compared due to their methodological standardization (12,13). However, it takes a certain amount of time for this to become common practice.

In considering the studies of mechanical fatigue on ceramic crowns, preparation design and substrate materials can vary. Epoxy resin filled with woven glass fibers is a commonly used dentin-like material (5-7,14). This material was first presented in 2010 as having similar elastic and adhesive properties to wet dentin (15). Despite its characteristics, studies have used other materials as a tooth substitute (8-11), such as ivory (16), acrylic resin (17,18), typodont (19), metal (20) and composite resins (21,22). However, it is known that there are differences in the failure modes obtained in different specimen forms. For this reason, the question arises whether epoxy resin and the presence of dental root (s) would influence the restoration’s mechanical properties. Few studies report the use of a dental preparation made of epoxy resin containing roots (5,23). Other variables are often simulated in laboratory tests, but have not been evaluated yet, which also lead to the following questions: should the substrate copy the tooth anatomy? Should a tooth be used or can a dentin-like material be used instead? Should the presence of the root (s) be considered? Or should the periodontal ligament be simulated?

Another point to consider is the stress distribution. If the distribution is not homogeneous, regions with stress peak values may concentrate and lead to failure of the material/structure (24,25). For evaluation of the stress distribution generated by masticatory loads in full crown restorations, finite element analysis (FEA) has been used (9,25) due to the ease of specimen standardization, low cost, and because it is a numerical method that offers an approximate solution (26). Thus, the goal of the present study was to evaluate the influence of substrate type (a dentin-like epoxy vs tooth), considering the presence of root (with and without) and the presence of periodontal ligament simulator (with and without) on the biomechanical behavior of full single monolithic crown restorations. The hypotheses were that: 1) the presence of the root, 2) the periodontal ligament, and 3) the type of substrate, would not interfere in the stress distribution in the restoration.

## Material and Methods

-Generation of the Geometric Models 

A 3D model of a sound tooth was scanned (InEos, Sirona Dental Systems GmbH, Bensheim, Germany) generating a stereolithographic file. The file was exported to CAD Rhinoceros software (Rhinoceros version 5.0 SR8, McNeel North America, Seattle, WA, USA). The generation of the geometric model followed the methodology in Dal Piva *et al.* (25), with similar characteristics. The model was replicated in five models for *in vitro* mechanical testing of molar crown simulation, according to the abutment preparation related in the literature ( Table 1). A preparation for a full-monolithic molar crown was designed with 5.5 mm of height and 12 degrees of occlusal convergence in the axial walls for all groups. Next, a 70-µm thick cement layer was created (27) between the intaglio surface of the restoration and the external bonding surface of the abutment.

**Table 1:**
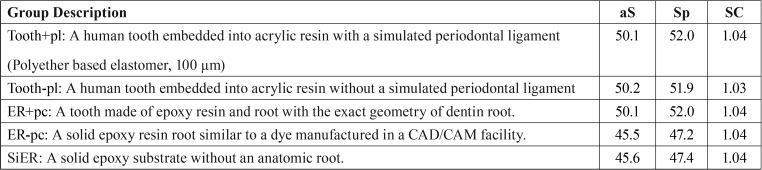
Group distribution according to the substrate design, average (aS) and stress peak values (Sp) in MPa and stress concentration factor (SC) obtained in the restoration.

-Finite element analysis (FEA)

**Figure 1:**
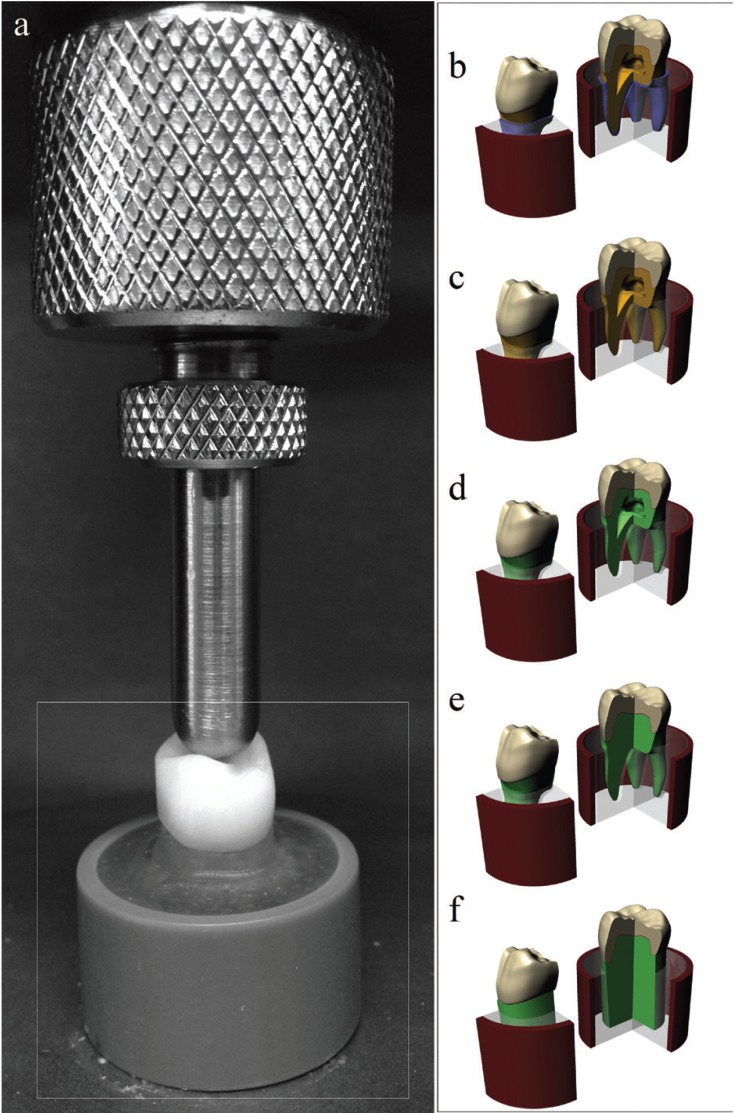
Illustration of the *in vitro* specimen and the modeling substrates design. A) In vitro specimen embedded in acrylic resin. B) Tooth+pl: A human tooth embedded into acrylic resin with a simulated periodontal ligament. C) Tooth-pl: A human tooth embedded into acrylic resin without a simulated periodontal ligament. D) ER+pc: A tooth made of epoxy resin and root with the exact geometry of dentin root. E) ER-pc: A solid epoxy resin root similar to a dye manufactured in a CAD/CAM facility. F) SiER: A solid epoxy substrate without an anatomic root.

Five different abutment preparations (Fig. 1) were obtained to determine the substrate influence on the crown behavior during a mechanical test. The crown model was defined to simulate zirconia reinforced lithium disilicate glass-ceramic [Vita Suprinity, Vita Zhanfabrick, Bad Säckingen, Germany, (E) = 70 GPa and Poisson ratio (ν) = 0.23] (28), embedded in the acrylic resin presented [E = 2.7 GPa and ν = 0.35] by a dentin root [ E = 18.6 GPa and ν = 0.32] or by an epoxy root [E = 18 GPa and ν = 0.30] (29). The incorporated resin cement had E = 6 GPa and ν = 0.30 (linear shrinkage = 2.7%) (27).

**Figure 2:**
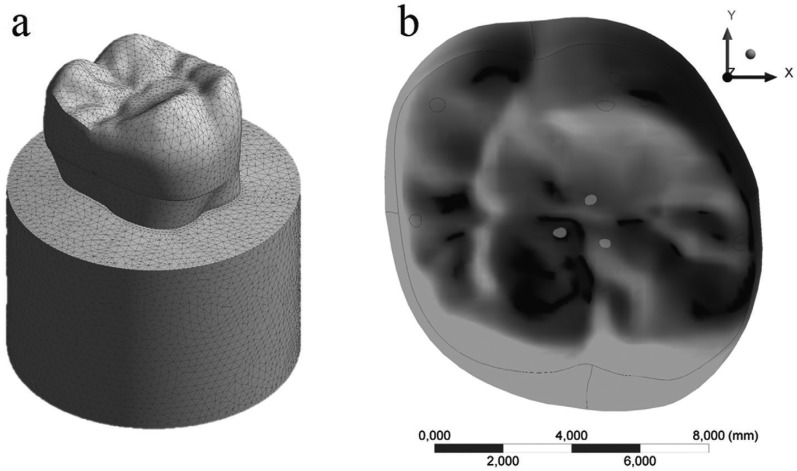
A) Mesh generation. B) 600-N load application area/points.

The models were imported through STEP format to the analysis software (ANSYS 17.2, ANSYS Inc., Houston, TX, USA), in which they were divided into mesh composed by nodes (mean value = 183,147, standard deviation = 22,189) and tetrahedral elements (mean value = 102,510, standard deviation = 10,256) (Fig. 2). The aspect ratio of mesh metrics presented average of 1.82 with a standard deviation of 0.49. Mechanical properties of each structure/material were inserted into the analysis software and each material was considered isotropic and homogeneous. Non-linear contact were considered as “Non-separated” between restoration/resin cement/tooth, in which the target and contact surfaces are tied for the remainder of the analysis, although sliding is permitted (26). The contact between the tooth and the fixation cylinder was considered perfectly bonded. Model fixation occurred at the base of the acrylic resin cylinder in all groups and an axial load of 600 N (32,34) was applied to the occlusal surface considering the tripoidism concept (26). A mesh convergence test (10%) was performed to guarantee that the mesh would not interfere in the results (30). To determine the average stresses, stress peaks and stress concentration factors, one hundred highest peaks were observed in each restoration.

## Results

**Figure 3:**
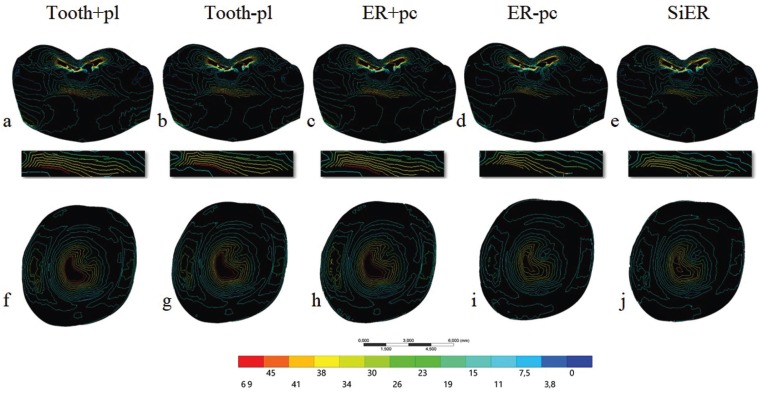
Maximum principal stress isolines in crown restoration sagittal view A-E) and in crown restoration intaglio surface F-J) according to the substrate design: Tooth+pl, Tooth-pl, ER+pc, ER-pc and SiER.

Maximum principal stresses were obtained for the crown, being the object of the study. Data was summarized through colorimetric isolines which allows the visualization of the limit between each stress fringe. Tensile stress (MPa) peaks were also recorded to determine the stress concentration factor, being calculated by the ratio of stress peak and average stress (25). Figure 3 is a sagittal view of the sectioned crown and demonstrates the decrease of red fringe and the consequent reduction of stress magnitude for epoxy-resin groups without pulp-chamber. The same behavior was observed for the crown intaglio surface showing the same stressed region, but with a decrease in the stress magnitude.  Table 1 demonstrates that the average stress and stress peaks were close for the restorations cemented onto Tooth+pl, Tooth-pl and ER+pc substrates. ER-pc and SiER substrates showed similar stress values between them, however they were 13% lower than the other groups. Considering the stress concentration factor, all groups showed similar results near 1.0, which means suitable stress distribution.

## Discussion

In an attempt to more accurately mimic the structures found in the oral environment and their micromovements, some authors have made their samples with recently extracted fresh human teeth and have also used an elastomeric material to make a synthetic periodontal ligament between the root and the inclusion resin (31-33). However, the results show that this laboratory step is not necessary for studies evaluating posterior monolithic crowns. Preparing the periodontal ligament is performed in two steps: first, the tooth is coated by a layer of removable material such as wax acting as a spacer between the dental root and the inclusion substrate; and in the second step after polymerization of the inclusion material, the wax gives space to the elastomer. The use of curved or divergent roots makes this procedure impossible, as it would be impossible to remove the root after polymerization of the resinous substrate around the roots. In addition, most commonly used materials to replace the periodontal ligament in laboratory studies are liquid silicone and polyether. These materials do not present any mechanical properties in common with the human periodontal ligament, since they have an approximately 10x greater elastic modulus (34), little or no adhesion to the dental substrate and minimal tear resistance. Since periodontal ligament simulation adds more laboratory steps during specimen preparation, restricts the use of teeth with favorable root anatomy for removal and insertion and does not contribute to render more reliable results to the oral findings, some authors began to ignore this step in their studies (35-37). Also, ligament confection can introduce bias into the study due to the different types of materials and their homogeneity, in addition to the thickness of the simulated ligament which can influence the obtained results. Thus, the direct inclusion of fresh human teeth enables using teeth with different anatomies and root forms and still uses the same human tissue found in the oral medium as substrate. These studies still depend on the approval of a research ethics committee to be conducted and the search for nearby anatomies is reported as part of the methodology. Other authors justify that they did not make the synthetic periodontal ligament due to the fact that other authors have not done so, while other authors do not omit the lack of this laboratory stage, but also do not discuss it. The results herein show that there is no difference in the tensile stress map for the crown in both sagittal or internal views in comparing the restorations in specimens with and without synthetic periodontal ligament. This finding is in agreement with previous studies (38,39) which did not simulate the periodontal ligament because there was no difference in the mechanical behavior of the restorations. In contrast, creating a synthetic periodontal ligament has been defended as being necessary to modify the fracture pattern of dental roots of anterior teeth (40), but this was not evaluated in this study.

In order to simplify the preparation of laboratory specimens even more, some authors have used similar materials to dental tissues as substrates for cementing the restorations. Among these materials, epoxy resin filled with woven glass fibers stands out as dentin-like material having scientific support (5-7,14,15). The simplification disregards the tooth anatomy, standardizes the samples dimension and shape (since the samples are machined) without the need for an ethics committee approval. By observing the results of the present study, it is possible to observe that the crown cemented on the epoxy-resin or tooth in fact behaves in a similar way, presenting the same map of tensile stresses with almost identical stress peaks. Thus, it can be estimated that a crown adhesively-cemented on an epoxy-resin or on a tooth (with or without synthetic periodontal ligament) will fail similarly due to the same axial force distribution.

One of the characteristics of the human tooth is the presence of the pulp chamber and the root canals that are filled with dental pulp (41). In general, studies that used synthetic substrates replacing a human tooth in the laboratory tests do not report the presence or absence of the pulp chamber space. Plus, there are no studies which evaluated the pulp chamber space influence on the resulting compressive forces in indirect restorations. Benazzi *et al.* (41) evaluated the effect of increasing or decreasing the pulp chamber space on the stress distribution in a healthy human tooth. But, when the increase of the pulp chamber space was simulated, anatomical alterations of the root dentin reduced the tensile stress concentration in the internal portion of the enamel and increased the stresses in the crown’s cervical third. There are reports on the dental remnant between restoration and pulp chamber, but the geometries were too simplified (often disks) (42,43).

One of the simulated groups in this theoretical study was a coronary preparation made in epoxy-resin without the pulp chamber or root canals, which would be the closest to a specimen machined from a solid resin cylinder. Figure 3 allow for verifying that the crown deforms less in the presence of an anatomical root without pulp chamber and also concentrates less tensile stress, suggesting a smaller possibility of failure for this type of specimen. In spite of this, the isolines in the colorimetric maps delimit the same tensile regions as in the other groups, but with smaller magnitude fringes. Thus, a specimen made in that model may take longer to fail or even fail at higher loads due to a reduction of approximately 10% of the maximum tensile values. However, the critical regions of possible fracture origin are the same in all evaluated groups. Finally, the complete simplification of the specimen using a dentin substitute without periodontal ligament, pulp chamber, root canals or anatomic roots is also possible (10). This is the most easily attainable specimen type due to its various simplifications, keeping only the crown anatomy as a similar geometry to that found in the oral environment. Regarding the biomechanical results, this specimen behaves like the previous one, decreasing the stress magnitudes due to the increased rigidity of the system, but allowing the same areas to be stressed during the test. A notable difference between stress peak and average stress values will result in values greater than 1 (32). All simulated subtracts herein showed similar stress concentration factors near to 1, suggesting a lower difference between average and peak values (i.e. homogeneous stress distribution). Thus, all evaluated subtracts showed very similar behavior.

When evaluating a laboratory specimen that simulates a posterior crown (Fig. 1), we often do not know the effects of various types of substrates on supporting the restoration. This theoretical manuscript aimed to gather all kinds of laboratory specimens that mimic an anatomical structure, as well as to show the simplifications which are often omitted or forgotten in order to challenge them and show what happens when the same load is applied in different specimens. Our goal was not to demean what has already been done, but rather to help and support specimen preparation in future studies. However, the theoretical results expressed herein should be extrapolated with caution, since the effects on anterior teeth, the results generated during oblique loading or results in the dental root were not considered. Moreover, the materials were considered isotropic, absent from defects and with homogeneous structures derived from the applied methodology.

From this study, it is possible to conclude that it is not necessary to make a synthetic periodontal ligament for investigations using molar full crowns, and the use of dental preparations may be performed with a dentine-like material without roots since the greater rigidity of the specimen is taken into account.
